# Bio-Ecological Features Update on Eleven Rare Cartilaginous Fish in the Central-Western Mediterranean Sea as a Contribution for Their Conservation

**DOI:** 10.3390/life11090871

**Published:** 2021-08-25

**Authors:** Antonello Mulas, Andrea Bellodi, Pierluigi Carbonara, Alessandro Cau, Martina Francesca Marongiu, Paola Pesci, Cristina Porcu, Maria Cristina Follesa

**Affiliations:** 1 Department of Life and Environmental Sciences, University of Cagliari, 09126 Cagliari, Italy; abellodi@unica.it (A.B.); alessandrocau@unica.it (A.C.); mfmarongiu@unica.it (M.F.M.); ppesci@unica.it (P.P.); cporcu@unica.it (C.P.); follesac@unica.it (M.C.F.); 2 CoNISMa Consorzio Nazionale Interuniversitario per le Scienze del Mare, 00196 Roma, Italy; 3 COISPA Tecnologia & Ricerca, 70126 Bari, Italy; carbonara@coispa.it

**Keywords:** cartilaginous fish, poorly-known species, age, reproduction, diet, Central-Western Mediterranean Sea

## Abstract

Cartilaginous fish are commonly recognized as key species in marine ecosystems for their fundamental ecological role as top predators. Nevertheless, effective management plans for cartilaginous fish are still missing, due to the lack of knowledge on their abundance, distribution or even life-history. In this regard, this paper aims at providing new information on the life-history traits, such as age, maturity, reproductive period, in addition to diet characteristics of eleven rare cartilaginous fish inhabiting the Central-Western Mediterranean Sea belonging to the orders Chimaeriformes (*Chimaera monstrosa*), Hexanchiformes (*Heptranchias perlo* and *Hexanchus griseus*), Myliobatiformes (*Aetomylaeus bovinus* and *Myliobatis aquila*), Rajiformes (*Dipturus nidarosiensis* and *Leucoraja circularis*), Squaliformes (*Centrophorus uyato*, *Dalatias licha* and *Oxynotus centrina*) and Torpediniformes (*Tetronarce nobiliana*), useful for their assessment and for future management actions. Particularly, the present paper provides for the first time the age estimation of *D. nidarosienis* and *L. circularis* which were both found capable of becoming older than ten years. In addition, the present study updates the sizes of first maturity of *C. uyato* and *D. licha*, which appeared to be capable of reproducing earlier than what was previously hypothesized, representing very valuable information for a better understanding of these rare species populations status and, eventually, their conservation. On the basis of the stomach content analysis, it was possible to identify five different predator groups.

## 1. Introduction

Marine ecosystems represent Earth’s largest biodiversity reservoirs [1], providing a wide range of contributions to human well-being, which collectively go under the name of Marine Ecosystem Services [2,3]. Due to this high resource availability, the human pressure on marine ecosystems is constantly rising, thus negatively impacting their biodiversity [4,5,6]. In this context, the Mediterranean Sea is considered, at the same time, a biodiversity hotspot and one of the most threatened marine areas in the world [7,8,9,10]. Even though the Mediterranean basin represents only 1% of the global oceans, it is estimated that it hosts up to the 18% of the world’s macroscopic marine species, about 30% of which are endemic [7]. Among these, cartilaginous fishes such as sharks, batoids (skates and rays) and chimaeras are certainly some of the most iconic species for the majority of public opinion, mainly due to their fundamental ecological role as top predators [11,12]. At the same time, due to their k-selected life-history traits, cartilaginous fish are particularly vulnerable to overfishing, habitat deterioration and to the modifications in the biological communities’ composition, which are also linked to biodiversity loss and to the introduction of alien species, in a rapidly changing scenario [13,14]. This led to a rapid decrease in abundance of many Mediterranean populations of sharks and batoids observed during the last 50 years and, in the worst cases, to some local extinctions [15,16,17]. The International Union for the Conservation of Nature (IUCN) estimates that at least 53% of the 88 species currently inhabiting the basin are Vulnerable, Endangered or Critically Endangered. Moreover, some of these assessments appear to be quite old and need to be updated. Furthermore, a large number of species (about 20%) are listed as Data Deficient [10], testifying that knowledge on them is insufficient to make any refined assessment, creating uncertainty on their extinction rates at a global level and excluding them from priority species list at a regional scale [18]. The underlying causes of this data scarcity are linked to several concomitant factors, such as low commercial value and taxonomic uncertainty [10,18,19,20,21,22]. Species with low commercial value, indeed, are not directly targeted by commercial fishery, being instead part of its by-catch. As a twofold consequence, on the one hand, these species are marketed grouped under generic names, raising additional difficulties in collecting landings information and, on the other, they often take second place in official data collection programs [19,22]. In addition, the problem is compounded by the lack of nomenclature stability and taxonomic resolution, due to the taxonomic issues typical of the cartilaginous species, combined to the ongoing changes in species occurrence in the different parts of the basin [10,23]. These problems are particularly severe for species characterized by low abundances or considered rare, for which only scattered observations and fragmented knowledge are available, thus resulting of extreme importance. From a conservation perspective, collecting all obtainable information on these species’ ecological and biological parameters, such as bathymetric and geographical range, maximum attained size and age, reproductive characteristics and feeding habits, is of fundamental importance to define different populations within the same regional areas and, more generally, to predict their vulnerability and extinction risk [18].

In this context, the aim of this work is to provide new and updated information on eleven cartilaginous fish species belonging to the orders Chimaeriformes (*Chimaera monstrosa*), Hexanchiformes (*Heptranchias perlo* and *Hexanchus griseus*), Myliobatiformes (*Aetomylaeus bovinus* and *Myliobatis aquila*), Rajiformes (*Dipturus nidarosiensis* and *Leucoraja circularis*), Squaliformes (*Centrophorus uyato, Dalatias licha*, *Oxynotus centrina*) and Torpediniformes (*Tetronarce nobiliana*), inhabiting the seas surrounding Sardinia (Central-Western Mediterranean). In general, all these species are considered rare or occasional (*sensu* [10]) in the Mediterranean Sea, showing a discontinuous distribution pattern in the different areas of the basin. In Sardinian waters, for their low or even null commercial value, they are not directly targeted by the commercial fishery, being part of the by-catch usually discarded at sea [17,24]. Since knowledge on many aspects of their life-history and ecology are still very limited and need to be updated, data on their size range, bathymetric distribution, age, reproduction and feeding habits, provided here, will increase the amount of information useful for their management and conservation.

## 2. Materials and Methods

### 2.1. Sampling

**Figure 1:**
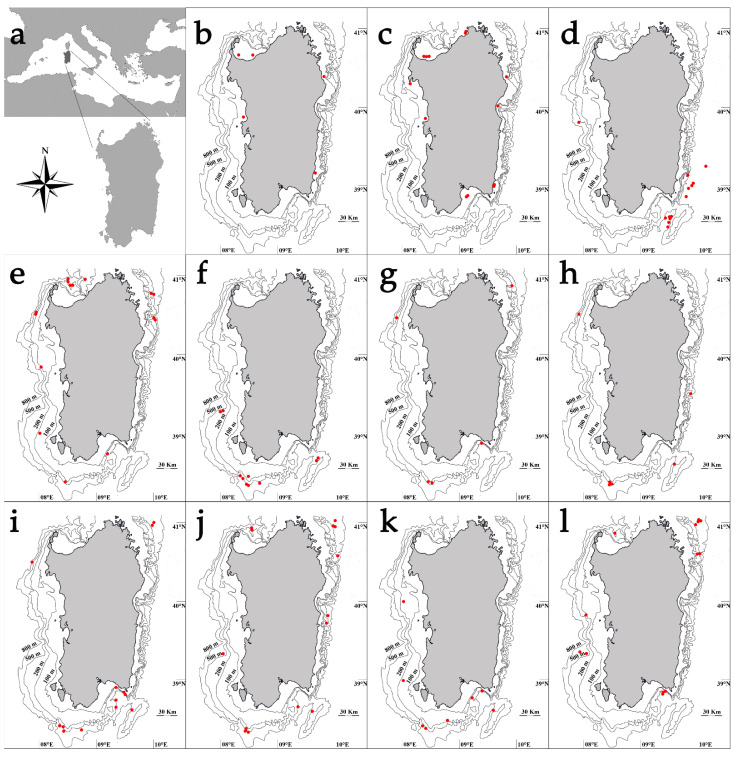
Investigated area (**a**) and sites of capture for: (**b**) *Aetomylaeus bovinus*; (**c**) *Myliobatis aquila*; (**d**) *Dipturus nidarosiensis*; (**e**) *Leucoraja cirularis*; (**f**) *Tetronarce nobiliana*; (**g**) *Heptranchias perlo*; (**h**) *Hexanchus griseus*; (**i**) *Centrophorus uyato*; (**j**) *Dalatias licha*; (**k**) *Oxynotus centrina*; (**l**) *Chimaera monstrosa*.

Specimens were collected between 2007 and 2021 in the seas around Sardinia (Central-Western Mediterranean Sea, FAO GFCM Geographical subarea 11) at depths ranging between 29 and 1573 m (Figure 1) during the experimental trawl surveys MEDITS (MEDiterranean International Trawl Survey; 102 hauls per year held during summer months; [25]) and the Deep Trawl campaign held by the University of Cagliari (119 hauls in total, equally distributed during the year). Additional samples were derived from the commercial fishery (24 fishing trips per year, equally distributed for seasons; Data Collection Framework program, EU Reg. 199/2008).

**Figure 2:**
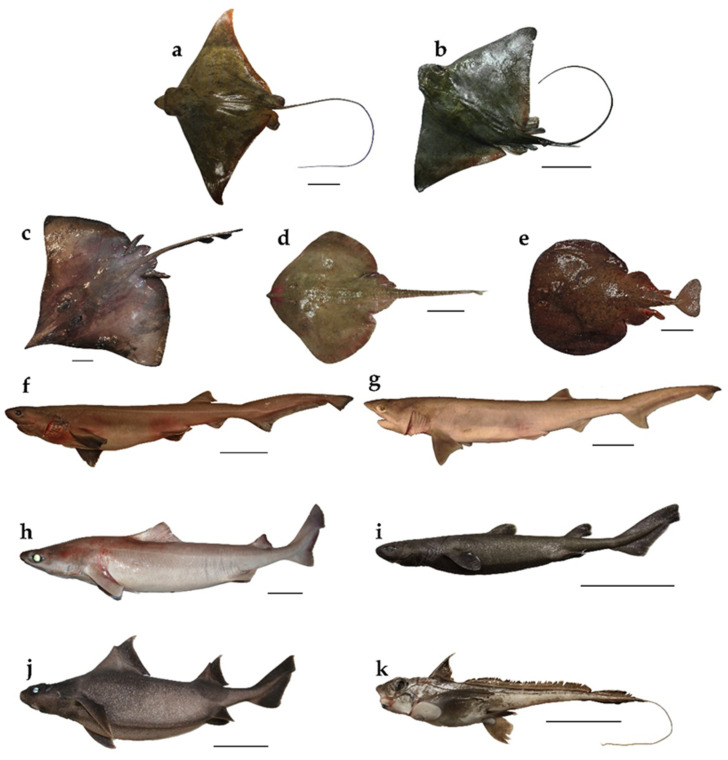
Studied species: (**a**) *Aetomylaeus bovinus*; (**b**) *Myliobatis aquila*; (**c**) *Dipturus nidarosiensis*; (**d**) *Leucoraja circularis*; (**e**) *Tetronarce nobiliana*; (**f**) *Hexanchus griseus*; (**g**) *Heptranchias perlo*; (**h**) *Centrophorus uyato*; (**i**) *Dalatias licha*; (**j**) *Oxynotus centrina*; (**k**) *Chimaera monstrosa*. Scale bars = 10 cm.

All specimens were weighed (Total Mass, TM, in g) and the main biometrics were recorded as follows: Total Length (TL, in cm) was used for the species belonging to the Torpediniformes, Rajiformes, Hexanchiformes and Squaliformes orders, while Disc Width (DW, in cm) and Pre-Ventral Length (SVL, in cm), were used, respectively, for Myliobatiformes and *Chimaera monstrosa* due to the fragile nature of the filamentous tail that characterize these species. Length–Mass relationships were calculated for the combined sexes according to the power curve function M = aL^b^, where M represents the Total Mass (TM) in g and L the main length used in cm (TL, DW and SVL depending on the species), a represents the intercept of the regression and b is the regression coefficient. The eleven studied species are shown in Figure 2.

### 2.2. Sex and Maturity Stage Estimation

Sex was registered and the maturity stage of each individual was determined following the scales proposed for Elasmobranchs by [26,27]. According to the scales, females belonging to viviparous species were classified into eight stages: (1) immature; (2) developing; (3A) capable of reproducing; (3B) early pregnancy; (3C) mid-pregnancy; (3D) late pregnancy; (4A) regressing; (4B) regenerating. Females belonging to oviparous species were classified into six stages: (1) immature; (2) maturing; (3A) mature; (3B) mature/extruding; (4A) resting; (4B) regenerating. Both viviparous and oviparous males were classified into five stages: (1) immature; (2) developing; (3A) capable of reproducing; (3B) actively spawning; (4) regressing (only for viviparous species) and (4A) resting (only for oviparous species). A Kolmogorov–Smirnov (K–S) test was performed in order to investigate possible differences in size between sexes for those species with a sufficient sample number [28].

For the most abundant species, ovarian fecundity, defined as the total number of yolked eggs, was estimated by counting the eggs in both ovaries of mature females (stages 3a–4a). Uterine fecundity, defined as the number of pups, was also recorded in females at mid- and late-term pregnancy (stages 3c–3d).

### 2.3. Age Estimation

In order to estimate specimens’ age, a portion of the vertebral column, including 8–10 *centra*, was extracted from the thoracic region of all species, with the exception of *A. bovinus*, *M. aquila* and *C. monstrosa*. To remove the exceeding soft tissues or muscles, after the removal of neural and haemal arches using a scalpel, each *centra* was soaked in a 5% hypochlorite solution for 5–10 min [29], depending on the *centra* dimension. With the aim of identifying the method that could allow the best band visibility, from a subsample of at least 30 individuals (15 females and 15 males of different size classes), 3 different *centra* per individual were selected and treated with two of the most common staining methods known in literature, Alizarine Red [30] and Silver Nitrate [31,32], and tested against the third *centra* kept unstained. The ageing process was carried out on vertebral *centra* sagittal sections following [33]. The section final thickness of 0.5 mm (±0.1 mm) allowed a better growth band visibility [34]. Vertebral sections (thickness 0.5 mm ± 0.1 mm) were obtained by grinding the whole *centra* embedded in a bi-component epoxy resin (Bio Optica, Technovit EPOX), grinding them using a polisher (ATM Saphir 320) equipped with progressively thinner abrasive discs [34,35]. Finally, age was obtained following the ageing methodology proposed for Elasmobranchs by [33].

### 2.4. Stomach Content Analysis

Stomachs were dissected and stored in a 5% formaldehyde solution. Then, contents were analyzed and classified to the lowest taxonomic level possible. The percent Frequency of Occurrence (%FO) of a specific prey item in all samples was calculated [12].

Data on *D. nidarosiensis*, collected in the period 2005–2011, and on *T. nobiliana,* collected between 2008 and 2019, have been presented in [36,37] and [38], respectively, while this study aims at updating the available information up to 2021.

## 3. Results

### 3.1. Myliobatiformes

#### 3.1.1. *Aetomylaeus bovinus*

**Figure 3:**
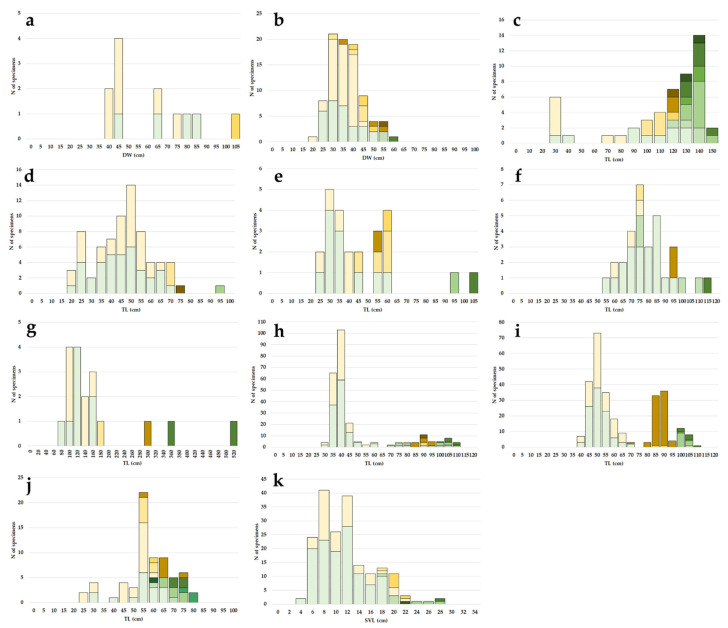
Size frequency distribution of the maturity stages of: (**a**) *Aetomylaeus bovinus*; (**b**) *Myliobatis aquila*; (**c**) *Dipturus nidarosiensis*; (**d**) *Leucoraja circularis*; (**e**) *Tetronarce nobiliana*; (**f**) *Heptranchias perlo*; (**g**) *Hexanchus griseus*; (**h**) *Centrophorus uyato*; (**i**) *Dalatias licha*; (**j**) *Oxynotus centrina*; (**k**) *Chimaera monstrosa*. 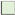 = *F1*; 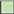 = *F2*; 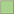 = *F3A*; 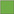 = *F3B*; 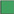 = *F3C*; 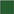 = *F3D*; 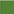 = *F4A*; 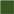 = *F4B*; 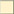 = *M1*; 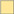 = *M2*; 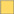 = *M3A*; 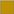 = *M3B*; 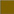 = *M4*.

The bull ray exhibited an extremely narrow bathymetric distribution, deeply related to the coastal line. A total of 12 individuals were caught at depths ranging from 35 to 50 m during the entire sampled period (mean ± s.d. = 42 ± 6.8 m) (Figure 1b). Specimens’ size ranged between 38.0 and 102.0 cm in DW (mean ± s.d. = 59.3 ± 21.0 cm) (Table 1), while weights (TM) oscillated between 481 and 13845 g (mean ± s.d. = 3665 ± 4032 g). Length–Mass relationship was M = 0.007 × L^3.149^ (r^2^ = 0.98) (Table 1). Sample was composed by four females whose sizes ranged between 41.3 and 81.0 cm in DW (mean ± s.d. = 66.8 ± 18.2 cm) and eight males ranging from 38.0 and 102.0 (mean ± s.d. = 55.5 ± 22.4 cm) (K–S, *p* > 0.05; Figure 3a). All specimens were found in the autumn and winter, and all were immature except one. The only one mature male was caught in winter (DW = 102.0 cm). Opisthobranch Gastropods (%FO = 100) and Anomuran Crustaceans (%FO = 75) (particularly *Paguristes eremita*) were present simultaneously and in large quantities in almost all the eight full stomachs analyzed.

#### 3.1.2. *Myliobatis aquila*

Similar to the bull ray, the common eagle ray also showed the narrowest bathymetric distribution among the analyzed species, being exclusively found in shallow coastal waters in the depth interval between 29 and 43 m (mean ± s.d. = 34 ± 3.0 m) (Figure 1c). The size of the 91 collected individuals ranged between 25.2 cm and 58.7 cm in DW (mean ± s.d. = 36.0 ± 73.6 cm) (Table 1), with TM between 220 and 5870 g (mean ± s.d. = 878 ± 920 g). Length-Mass relationship was M = 0.011 × L^3.076^ (r^2^ = 0.93) (Table 1). Females (N = 33) size range, included between 26.0 and 58.7 cm in DW (mean ± s.d. = 36.8 ± 8.6 cm), was similar to that observed in males (N = 58), whose values ranged between 25.2 and 50.4 cm in DW (mean ± s.d. = 35.5 ± 6.5 cm) (K–S, *p* > 0.05; Figure 3b). Almost all the individuals sampled across the seasons were immature. A few mature males (DW = 31.0–50.2 cm) were found in all seasons except in winter, whereas a few post spawning regressing females (DW = 58.7 cm) were caught only in winter and spring. Gastropods (%FO = 100) and Anomuran Crustaceans (%FO = 82) (mainly *Paguristes eremita*) represented the main prey items found in the 11 full stomachs examined, followed by Sipunculids (%FO = 36), such as *Aspidosiphon muelleri*, and Polychaetes (%FO = 25).

### 3.2. Rajiformes

#### 3.2.1. *Dipturus nidarosiensis*

**Figure 4:**
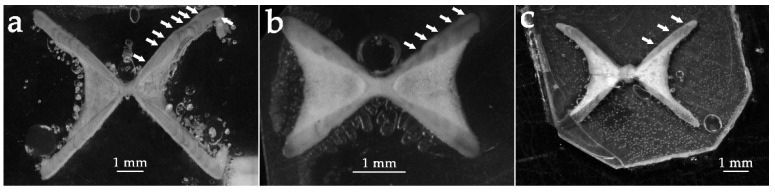
Examples of vertebral *centra* thin sections. (**a**) *Dipturus nidarosiensis* (TL = 133.5 cm, female); (**b**) *Leucoraja circularis* (TL = 51.7 cm, male); (**c**) *Tetronarce nobiliana* (TL = 53.2 cm, female). White arrows indicate the true winter rings.

*Dipturus nidarosiensis* revealed a bathymetric distribution strongly pointed to deep bottoms, reporting one of the deepest bathymetric ranges (550–1573 m; mean ± s.d. = 905 ± 332.4 m) (Figure 1d). All specimens (N = 47) were caught between 2005 and 2017. The samples’ TL ranged between 24.0 and 148.2 cm (mean ± s.d. = 111.9 ± 31.3 cm) (Table 1), with TM between 38 and 13783 g (mean ± s.d. = 7542 ± 3904 g). Length–Mass relationship was M = 0.002 × L^3.220^ (r^2^ = 0.97) (Table 1). Additionally, this species seemed to exhibit a strong sexual dimorphism with females growing bigger than males, with 379.0–1482.0 mm TL (mean ± s.d. = 123.2 ± 20.7 cm) compared to 24.0–1190.0 cm TL (mean ± s.d. = 85.2 ± 36.2 cm) (K–S, *p* < 0.05; Figure 3c). The vertebral *centra* (Figure 4a) ageing estimation seemed to indicate a medium/long life span for this species, being that the oldest female (TL = 148.2 cm) and male (TL = 118.0 cm) were both estimated to be 12 years old. The species showed an extended reproductive cycle since immature, mature (females’ TL = 131.0–138.5 cm; males’ TL = 112.0–119.0 cm) and post spawning (TL = 128.4–140.5 cm) individuals were found in all seasons. The majority of extruding females (TL= 129.5–137.6 cm) were found in summer. A clear bathymetric segregation by maturity stage was found with mature females more common in upper deep waters (between 550 and 1000), and immature individuals at lower depths (1145–1593 m). The analysis of 15 full stomachs showed that the diet was strongly based on the Brachyuran Crustacean *Geryon longipes* which represented the main prey item (%FO = 75), while other Elasmobranchs (%FO = 25), Actynopterygii (%FO = 17), Polychaetes (%FO = 17) and Cephalopods (%FO = 8) completed the alimentary spectrum.

#### 3.2.2. *Leucoraja circularis*

In the period between 2008 and 2020, 77 sandy ray specimens were found in sea bottoms from 128 to 620 m (mean ± s.d. = 382 ± 140.8 m) (Figure 1e). Specimens’ size ranged between 17.1 and 90.3 cm in TL (mean ± s.d. = 46.1 ± 16.1 cm) (Table 1) with weights of 17 to 3917 g (TM) (mean ± s.d. = 711 ± 922 g). Length–Mass relationship was M = 0.001 × L^3.360^ (r^2^ = 0.99) (Table 1). Conversely to what was observed in some of the other analyzed species, females and males presented no major difference in size (K–S, *p* > 0.05). Females (N 42) ranged between 17.1 and 90.3 cm TL (mean ± s.d. = 46.9 ± 17.6 cm), while males’ (N = 35) TL varied between 19.8 and 73.4 cm (mean ± s.d. = 45.0 ± 14.4 cm) (Figure 3d). However, females were found able to reach much higher weights than males, at 17–3917 g (TM) (mean ± s.d. = 631 ± 751 g) and 25–1872 g (TM) (mean ± s.d. = 541 ± 494 g), respectively. The analysis of vertebral *centra* thin sections (Figure 4b) revealed the species as characterized by a potentially medium/long life span. The oldest male and female individuals (TL = 73.4 and 76.6 cm, respectively) were both estimated to be 11 years old. Unfortunately, it was not possible to estimate the age of the biggest individual caught (90.3 cm TL). Only two of the caught specimens, both collected in June, were found mature, precisely corresponding to the biggest female (90.3 cm TL) classified as capable of reproducing, and to the biggest male (73.4 cm TL) which was in regressing stage. The prey items found in the 19 full stomachs belonged to four main groups: Crustaceans, which represented the most important (%FO = 63), followed by Actinopterygians, other Elasmobranchs and Cephalopods (%FO = 53, 21 and 21, respectively).

### 3.3. Torpediniformes

#### *Tetronarce* *nobiliana*

During the period between 2009 and 2021, 26 great torpedo ray individuals (13 females and 12 males) were captured. The specimens were caught at depths between 200 and 600 m (mean ± s.d. = 489 ± 127.8 m) (Figure 1f). While the minimum observed size recorded was similar for females (TL = 24.2 cm; TM = 222 g) (Table 1) and males (TL = 24.0 cm; TM = 211 g) (Table 1), the biggest female (TL = 101.5 cm) was nearly twice the size (TL = 66.6 cm) and weighed almost three times more (female TM = 15130 g versus male’s TM = 48 g) than the biggest male. The mean sizes (±s.d.) recorded were 44.9 ± 25.7 cm (TL) and 2830 ± 4923 g (TM) for females and 44.6 ± 14.5 cm (TL) and 1842 ± 1550 g (TM) for males (K–S, *p* > 0.05; Figure 3e). Length–Mass relationship, calculated for the mixed sexes, was M = 0.014 × L^3.026^ (r^2^ = 0.99) (Table 1). The direct age estimation, carried out on sectioned vertebral *centra* (Figure 4c), revealed that females were capable of growing older than males, attaining 17–18 years and 9 years, respectively. Only two mature females were found, one was recognized as capable of reproducing (TL = 98.0 cm, caught in January), the other as regressing (TL = 101.5 cm, caught in April). Concerning males, three mature specimens were collected, two of them were capable of reproducing (TL = 66.6 cm caught in July and TL = 59.3 cm caught in April) while the other was active (TL = 54.0 cm, caught in February). Actinopterygii (%FO = 100) represented the only prey item found in *T. nobiliana*; the presence of remains of bony fishes were recorded in every analyzed stomach.

### 3.4. Hexanchiformes

#### 3.4.1. *Heptranchias perlo*

In the period from 2009–2021, a total of 31 sharpnose sevengill shark individuals was collected. All specimens, ranging from 54.5 to 113.3 cm TL (mean ± s.d. = 77.5 ± 14.0 cm) (Table 1) and 94 to 4255 g TM (mean ± s.d. = 1553 ± 884 g), were caught at depths between 336 and 600 m (mean ± s.d. = 585 ± 152.8 m) (Figure 1g). Length–Mass relationship was M = 0.004 × L^2.924^ (r^2^ = 0.94) (Table 1). The sample was constituted almost entirely of females (N = 25) which ranged between 54.5 and 113.3 cm TL (mean ± s.d. = 78.0 ± 14.2 cm). Males (N = 6) were slightly smaller than females, ranging between 57.1 and 95.0 cm TL (mean ± s.d. = 75.4 ± 14.4 cm), although this difference was not statistically significant (K–S, *p* > 0.05; Figure 3f). Vertebral *centra* appeared poorly calcified in this species and none of the tested staining techniques were able to enhance band visibility. Immature individuals (mainly females) were caught across the seasons. Only three individuals were mature: the two largest males (TL = 90.4 and 95.0 cm), caught in spring and summer, respectively, were active spawners, and the largest female (TL = 113.3 cm), caught in summer, was regressing. Out of the 31 stomachs analyzed, 13 were full. The main prey categories were Cephalopods (%FO = 8), and particularly Ommastrephids (%FO = 65) such as *Todarodes sagittatus*, Actinopterygii (%FO = 65), and other Elasmobranchs (%FO = 12), such as *Etmopterus spinax* and *Galeus melastomus*.

#### 3.4.2. *Hexanchus griseus*

A total of 19 bluntnose sixgill shark specimens from 75.9 cm TL (TM = 1673 g) to 518.0 cm TL (estimated TM = 400,000 g) was collected from 2009 and 2021 at depths between 500 and 624 m (mean TL ± s.d. = 162.7 ± 115.9 cm; mean TM ± s.d. = 61450 ± 126881 g; mean depth ± s.d. = 564 ± 46 m) (Table 1; Figure 1h). Length–Mass relationship was M = 0.002 × L^3.117^ (r^2^ = 0.99) (Table 1). Females’ (N = 10) TLs were between 75.9 and 518.0 cm (mean ± s.d. = 176.9 ± 144.4 cm), while males’ (N = 9) ranged from 83.1 to 299.0 cm (mean ± s.d. = 145.0 ± 69.0 cm) (K–S, *p* > 0.05; Figure 3g). Similar to what was observed in the sharpnose sevengill shark, due to the extremely poor calcification level of the vertebrae, none of the staining methods applied to the *centra* allowed revealing a clear growth band pattern. Immature individuals (N = 15) of both sexes were found across the year. Only the largest male and female were mature: the male (TL = 299.0 cm), caught in summer, was an active spawner, while the female (TL = 518.0 cm) was at regressing stage and caught in winter (Figure 3g). Ten out of the nineteen stomachs analyzed were empty. Inside the remaining nine, large Actinopterygii (%FO = 83), such as *Xiphias gladius*, *Merluccius merluccius* and *Lepidopus caudatus*, Ommastrephid Cephalopods (%FO = 33) such as *T. sagittatus* and *Loligo forbesii,* and Cetacean remains (%FO = 17) were found.

### 3.5. Squaliformes

#### 3.5.1. *Centrophorus uyato*

A total of 285 gulper sharks were collected in the period 2009–2020 between 360 and 653 m depth (mean ± s.d. = 527 ± 60.9 m) (Figure 1i). Specimens ranged from 38.3 to 106.5 cm in TL (mean ± s.d. = 62.6 ± 19.7 cm) (Table 1) and 224 to 7151 g in TM (mean ± s.d. = 1764 ± 1839 g). Length–Mass relationship was M = 0.001 × L^3.344^ (r^2^ = 0.99) (Table 1). Females were lower in number and attained larger sizes (N = 122; TL = 38.3–106.5 cm; mean ± s.d. = 57.5 ± 20.2 cm) than males (N = 163; TL = 38.3–91.7 cm; mean ± s.d. = 64.8 ± 18.5 cm) (K–S, *p* < 0.05; Figure 3h). From a subsample of 30 specimens (equally distributed by sexes and sizes), vertebral *centra* were extracted and stained with all the above-mentioned techniques, however, none of them permitted the growth band identification with a reasonable confidence level. Immature individuals were predominant in all seasons. Mature males (ready to mate, TL = 67.8–92.5 cm) were caught across the year, but mature females (TL= 96.5–103.6 cm) and those in post-partum (TL = 98.3–106.5 cm) were sampled to a lesser extent in autumn, winter and spring (Figure 3h). The preferential preys, found inside 91 full stomachs, were Actinopterygii (%FO = 68) and Ommastrephid Cephalopods (%FO = 30), such as *Illex coindetii*, *Todaropsis eblanae*, *T. sagittatus*, and *L. forbesii*, while other Elasmobranchs, and Decapod and Euphasiid Crustaceans were preys of secondary importance (%FO = 8, 8 and 6, respectively).

#### 3.5.2. *Dalatias licha*

From 2008 to 2021, 251 kitefin sharks, ranging between 26.2 and 108.6 cm TL (mean ± s.d. = 46.9 ± 21.8 cm) (Table 1) and 71 to 7749 g TM (mean ± s.d. = 860 ± 1533 g) were caught at depths between 510 and 682 m (mean ± s.d. = 533 ± 60.3 m) (Figure 1j). Females (N = 144) outnumbered males (N = 107), but the individuals of the two sexes were similar in size (K–S, *p* > 0.05): females’ TLs were between 28.9 and 108.6 cm (mean ± s.d. = 47.4 ± 22.9 cm) while males ranged from 26.2 to 94.4 cm TL (mean ± s.d. = 46.3 ± 20.2 cm) (Figure 3i). Length–Mass relationship for the mixed sexes was M = 0.002 × L^3.176^ (r^2^ = 0.96) (Table 1). None of the staining methods applied on a subsample of 30 specimens (equally distributed by sexes and sizes) appeared to allow the identification of the different growth bands. Immature individuals were prevalent in all seasons. Few mature specimens of both sexes were caught throughout the year. In particular, mature females (TL = 98.0–103.0 cm) were caught in all seasons, while those in early pregnancy (TL = 104.3–106.0 cm) were caught in summer and post-partum (TL = 104.2–108.6 cm) during all seasons except summer. Mature and especially active males (TL = 82.8–94.4 cm) were sampled all throughout the year, but those in the regressing phase (TL = 86.5–89.5.5 cm) only in winter and spring. Ovarian fecundity, registered for five mature females, ranged between night to ten yolked eggs. The diet, studied through the analysis of 106 full stomachs, was based on a wide variety of prey items, among which the most important were Actinopterygii (%FO = 53), mainly Myctophids and Macrourids (%FO = 14 and 8, respectively), Cephalopods (%FO = 45) (particularly Sepiolids and Ommastrephids; %FO = 24 and 22, respectively), other Elasmobranchs (%FO = 36), such as *Etmopterus spinax* and Decapod and Euphasiid Crustaceans (%FO = 30 and 17, respectively).

#### 3.5.3. *Oxynotus centrina*

Between 2007 and 2021, 67 specimens from 22.7 to 78.2 cm TL (mean ± s.d. = 54.9 ± 12.6 cm) (Table 1) and from 71 to 6767 g TM (mean ± s.d. = 2175 ± 1640 g) were collected at depths between 180 and 600 m (mean ± s.d. = 514 ± 49 m) (Figure 1k). Length–Mass relationship was M = 0.001 × L^3.699^ (r^2^ = 0.95) (Table 1). Females (N = 31) ranged from 25.6 cm to 78.2 cm TL (mean ± s.d. = 59.9 ± 12.8 cm), while males were from 22.7 to 74.6 cm TL (mean ± s.d. = 50.6 ± 10.9 cm) (K–S, *p* < 0.05; Figure 3j). Due to the particularly poor calcification level of this species vertebral *centra*, none of the tested staining techniques were able to enhance band visibility. Immature individuals were sampled during all seasons, while active males (TL = 53.5–74.6 cm) were caught in winter and summer. Mature females (TL = 60.8–72.2 cm) were found throughout the year; in particular females in mid-pregnancy (TL = 71.5–78.2 cm) were sampled in winter and spring and the only specimen in late pregnancy (TL = 60.0 cm) in May. Females in post-partum (TL = 65.5–74.3 cm) were caught in winter and spring. Ovarian fecundity ranged from 10 to 12 ripe oocytes in mature females, while uterine fecundity was from 13 to 16 embryos. Although all analyzed stomachs contained liquid, the only prey items found in 11 full stomachs were embryos of other Elasmobranchs (%FO = 18), particularly Scyliorhinids and Rajids.

### 3.6. Chimaeriformes

#### *Chimaera* *monstrosa*

A total of 192 rabbitfishes were collected between 2009 and 2021 at depths from 400 to 1212 m (mean ± s.d. = 457 ± 100.8 m) (Figure 1l). The specimens’ size ranged from 3.6 to 27.8 cm SVL (mean ± s.d. = 10.7 ± 4.7 cm) (Table 1), while the weight was between 5 and 1197 g (mean ± s.d. = 116 ± 170 g). Length–Mass relationship was M = 0.066 × L^2.941^ (r^2^ = 0.97) (Table 1). Females (N = 132), which ranged in size from 3.6 to 27.8 cm SVL (mean ± s.d. = 10.6 ± 4.6 cm), outnumbered males (N = 60), which sizes were between 4.35 and 21.8 cm SVL (mean ± s.d. = 11.1 ± 4.9 cm) (K–S, *p* > 0.05; Figure 3k). The majority of the individuals sampled across the seasons were immature individuals of both sexes. Few mature females (SVL = 22.9–27.8 cm) were found in spring and summer, one at regressing phase (SVL = 26.5 cm) in autumn and one in regenerating stage (SVL = 21.3 cm) in summer. Mature males (SVL = 18.2–21.8 cm) were sampled in winter and summer. *C. monstrosa* females segregated by maturity stage. In fact, mature specimens were more common in upper deep waters, while immature individuals were observed at greater depths and developing ones at shallower depths. The diet, analyzed from 26 full stomachs, was based mainly on Crustaceans (%FO = 76), particularly Brachyurans (%FO = 32) and Amphipods (%FO = 32), and Bivalve Mollusks (%FO = 72).

## 4. Discussion

The present study aimed to enrich the available literature on rare and poorly-known cartilaginous species, also taking advantage of the relatively high abundance of these species in Sardinian waters [17,24], providing information on some of their life-history traits, such as age, maturity, reproductive period, in addition to diet features. This kind of information has also been recognized as extremely useful in consideration of the global IUCN Red List reassessment, which recently started from the Northeast Atlantic and Mediterranean Sea cartilaginous fish species [18]. Indeed, taking into account the extreme data deficiency and general lack of information characterizing these species, this new assessment was highly focused on trait-based predictions using biological and ecological trait data [18]. For example, the main traits associated with a higher extinction risk were large body size, small bathymetric and geographic range, and ecological specializations (such as a narrow trophic niche) [18]. With respect to the existing data on the species analyzed, this work represents a useful update toward a more comprehensive assessment and, consequently, an improvement for their conservation status.

### 4.1. Bathymetric and Geographic Distribution

Some bathymetric ranges have been updated with respect to what is reported in literature. The species belonging to the Myliobatiformes order, for example, exhibited a much narrower bathymetric distribution in Sardinian waters, being always found in the first 50 m of the water column, while in literature their range is extended up to 150 m for *A. bovinus* [39,40] and to 573 m for *M. aquila* [41]. This fact could indicate, for these species, a higher degree of vulnerability than previously estimated, particularly at the regional level. The shallower waters are, indeed, the most exploited by artisanal fishery and in general represent the marine areas most impacted by a large amount of anthropic activities. In this regard, presenting such a narrow bathymetric range limited to this particular area could represent an additional level of threat for these species’ conservation status. Despite the relative rarity of *C. monstrosa*, 47 of the total 192 individuals were caught together in a single commercial fishing haul that occurred on 24 July 2020 (38°50′ N; 9°38′ E). Most of these specimens were immature and quite small (mean SVL = 13.1 cm). This peculiar aggregation might indicate the presence of an essential habitat for the species, such as a juvenile feeding ground or even a nursery area. Unfortunately, the data collected during a single haul do not consent to verify properly the latter assumptions. However, considering that the designation of essential fish habitats is one of the main goals that environmental managers are facing [42,43], and given the obvious implications for the species conservation, further studies must be endorsed to investigate if the area may represent a sort of sanctuary or a nursery ground for the species *sensu* [44]. 

### 4.2. Size Range

According to [45,46] the maximum reported size for *H. griseus* was 482 cm (TL) even though both studies hypothesized that the species can probably reach Total Length of about 550 cm. In this regard, the observed size of the biggest specimens caught in Sardinian Seas (518 cm TL) seems to endorse the hypothesized maximum size proposed by these authors. In consideration also of the records of two large specimens described by [47], with TL 523 cm and 600 cm, caught in the South-East Aegean Sea and the Sea of Marmara, respectively, the largest Sardinian specimen appears to be the third largest bluntnose sixgill shark ever recorded and consequently the largest in Western Mediterranean Sea.

A strong size-related sexual dimorphism (SSD), with females attaining bigger sizes than males, is quite often reported in sharks (e.g., [37,48,49,50,51]) and batoids (e.g., [35,52,53]). Most of the species analyzed in the present study seemed to follow this trend, except for both eagle ray species *M. aquila* and *A. bovinus,* which exhibited no signs of SSD, representing an apparent exception in Cartilaginous fish biology. Certainly, the low sample number, especially regarding *A. bovinus*, may have affected this outcome. However, considering that similar findings have been reported for other Myliobatiformes (e.g., the white-spotted eagle ray *Aetobatus narinari*, [54]), the hypothesis of no SSD in these Mediterranean species seems to be plausible. 

### 4.3. Age and Growth

Information on a certain species age and growth are generally considered essential to a proper assessment [53]. In this regard, the present paper provides the very first age data, although preliminary, on the *D. nidarosiensis* and on *L. circularis* since, accordingly to [55] for the latter species, “Age at maturity, longevity, size at birth, reproductive age, gestation time, reproductive periodicity and fecundity are unknown”. Growth bands on both species vertebral *centra* thin sections appeared relatively easy to be recognized, although in *D. nidarosiensis*, similarly to its congeneric *D. oxyrinchus* [35], the presence of growth bands split in doublets or triplets have been detected. For this reason, the age estimation on this species could be tricky and must be carefully evaluated. Both skates’ species seemed capable of reaching relatively old ages, and the maximum observed age in *L. circularis* appeared highly consistent with the longevity reported for its congeneric *L. naevus* [56]. The relatively high longevity showed by these skates contributes to indicating them as species particularly prone to conservation risks, *sensu* [18], thus highlighting the need to further investigate this aspect, considering also the high reliability of the age estimation protocol employed in this study [33,57].

Indeed, the age estimation analysis showed vertebral *centra* thin sections as a highly consistent and easily interpretable structure for ageing purposes on the analyzed species belonging to the Rajiformes and Torpediniformes orders. Conversely, the ageing protocol was not able to allow any attempt of age determination of the deep-sea sharks belonging to the Hexanchiformes and Squaliformes orders, since none of the applied staining methods permitted to enhance the contrast between growth bands. This issue appears to be related to the extremely low calcification level of this species skeleton, already documented in species such as *H. griseus* [58]. Considering the important role that age and growth data cover in species management and conservation and the species-related effectiveness of staining methods [29,58], future studies testing new methods on poorly-known species should be endorsed. Information regarding species age and growth are generally recognized as difficult to achieve, especially for cartilaginous species, which often present additional issues with respect to bony fish [38]. One of these problems is the lack of age validation studies, such as mark-recapture campaigns that are extremely subject to a variety of issues, e.g., tagging-related mortality, tag loss and tag detection and reporting rates. These problems are further worsened by the low recapture probability, linked to the rarity of the species [57]. For this reason, most of the studies did not validate the band deposition periodicity in vertebral centra and even fewer the absolute age [57]. The majority of the studies, indeed, are only based on the annual patterns of growth areas in the form of a succession of opaque and transparent bands that are assumed to be *annuli*, as in the present work. In this regard, given the data paucity characterizing these species, every kind of information results are crucial.

### 4.4. Reproductive Features

Concerning reproductive features, the present paper updated the available information on some of the analyzed species, such as the reproductive period and the size of the smallest mature individuals. Regarding the Hexanchiformes order, an active *H. perlo* male smaller than the reported first maturity size [21] has been found in the present paper. Additionally, mature males below the reported first maturity size have been observed also in the Squaliformes *C. uyato*, whereas this situation in *D. licha* emerged in both sexes [21]. Apparently, the present study updated the sizes of first maturity of these species. All of them appeared to be capable of reproducing earlier than what was previously hypothesized. Although, due the rareness of some of the analyzed species a rather scattered observation pattern was returned, generally, a continuous breeding cycle throughout the year can be hypothesized. The last statement seems to fit particularly well to the analyzed species that inhabits deeper water, confirming the reproductive pattern of deep-water cartilaginous fishes commonly reported, e.g., [34,51] and references therein. These results may represent very valuable information for a better understanding of these rare species’ populations status and, eventually, their conservation.

### 4.5. Diet

The stomach content analysis showed that the studied species could be gathered in five different groups on the basis on their main prey items. The first group, constituted by Myliobatiformes, was defined by the high abundance of gastropods and hermit crabs in the diet. It appeared interesting to note how, despite the peculiar mouth structure of eagle rays, particularly adapted in crushing shells, our results showed *A. bovinus* to feed mostly on soft-bodied gastropods, such as Opisthobranchs. This result could represent for the species a strategy of avoiding competition with *M. aquila* that share the same habitats. The second group included the Rajiformes and *C. monstrosa*, which seemed to share a preference for Crustaceans, showing sometimes a high preponderance for prey, such as the large deep-water crab *G. longipes* in *D. nidarosiensis*. Our data represent the very first information on the *L. circularis* diet and a huge update on the Norwegian skate in the Mediterranean Sea. *T. nobiliana* constituted another separated group; its sit and wait predatory habit, in addition to its capacity to stun even large preys, led to a high importance of bottom-related bony fishes in the diet [38]. The fourth group comprised deep-water sharks belonging to Hexanchiformes, both *H. perlo* and *H. griseus*, and the Squaliformes *D. licha* and *C. uyato*, which fed on relatively large and fast-moving preys such as bony fishes, squid-like Cephalopods and, in *D. licha* and *H. perlo*, other Elasmobranchs. Finally, *O. centrina*’s feeding habits seem focused on other Elasmobranchs’ egg capsules, due to their stomachs only having the presence of yolk-like liquid and other Elasmobranchs’ embryos. These findings appeared in disagreement with the worm-eating description of this species proposed by [59], but in line with [60], who identified *O. centrina* as an egg-case predator. In this regard, new studies based on molecular analysis are needed to clarify this peculiar aspect. In addition to the large body size and bathymetric distribution [18], particular feeding habits could also play an important role in defining species vulnerability.

In conclusion, the present paper provided new, and in some cases the first, information regarding reproduction, growth and feeding behavior of eleven rare and poorly- known Elasmobranch species. This kind of information represents a solid step toward a better comprehension of the life-history of these species and, given the unavoidability of these data for any effective management plan, an extremely valuable baseline for future conservation action on these key species for ecosystem equilibrium.

## Figures and Tables

**Table 1:** Summarized information on IUCN classification and year of assessment, number of individuals collected (N), size range, Length–Mass relationship parameters (a, b and r^2^), maximum observed age, mature specimens period and number of observations (Nm) and main diet items of the eleven studied species. CR = Critically Endangered; DD = Data Deficient; EN = Endangered; LC = Least Concern; NA = Not Assessed; NT = Near Threatened; VU = Vulnerable.

	IUCN Assessment				Length-Mass Relationship						Mature Specimens		
Species	Global	Mediterranean	N	Depth Range (m)	a	b	r^2^	Sex	Size Range (cm)	Maximum Observed Age (Years)	Period	Nm	Diet Items
*A. bovinus*	CR (2020)	CR (2016)	12	35–50	0.007	3.149	0.98	F	41.3–81.0 *		ND	-	Gastropods, Hermit crabs
								M	38.0–102.0 *		February	1	
*M. aquila*	CR (2020)	VU (2016)	91	29–43	0.011	3.076	0.93	F	26.0–58.7 *		March, June	2	Gastropods, Hermit crabs
								M	25.2–50.4 *		June–December	8	
*D. nidarosiensis*	NT (2014)	NA	47	550–1573	0.002	3.220	0.97	F	379–1482	12	January–December	34	Crustaceans
								M	24.0–1190.0	12	January–December	4	
*L. circularis*	EN (2014)	CR (2016)	77	128–620	0.001	3.360	0.99	F	17.1–90.3	11	June	1	Crustaceans
								M	19.8–73.4	11	June	1	
*T. nobiliana*	DD (2004)	LC (2016)	26	200–600	0.014	3.026	0.99	F	24.2–101.5	17/18	January, April	2	Bony fish
								M	24.0–66.6	8	ND	-	
*H. perlo*	NT (2019)	DD (2016)	31	336–600	0.004	2.904	0.94	F	54.5–113.3		June	1	Bony fish, Cephalopods
								M	57.1–95.0		March	2	
*H. griseus*	NT (2019)	LC (2016)	19	500–624	0.002	3.117	0.99	F	75.9–518.0		January	2	Bony fish, Cephalopods
								M	83.1–299.0		July	1	
*C. uyato*	EN (2019)	NA	285	360–653	0.001	3.344	0.99	F	38.3–106.5		January–December	20	Bony fish, Cephalopods
								M	38.3–91.7		January–December	77	
*D. licha*	VU (2017)	VU (2016)	251	510–682	0.002	3.176	0.96	F	28.9–108.6		January–December	13	Bony fish, Cephalopods
								M	26.2–94.4		January–December	18	
*O. centrina*	VU (2007)	CR (2016)	67	180–600	0.001	3.699	0.95	F	25.6–78.2		January–December	14	Elasmobranchs Egg-capsules
								M	22.7–74.6		January–March, July	7	
*C. monstrosa*	VU (2019)	NT (2016)	192	400–1212	0.066	2.941	0.97	F	3.6–27.8 **		May, July	2	Crustaceans, Bivalves
								M	4.4–21.8 **		January, July	5	

* Disc Width; ** Pre-Ventral Length.

## References

[1] Appeltans W., Ahyong S.T., Anderson G., Angel M.V., Artois T., Bailly N., Bamber R., Barber A., Bartsch I., Berta A. (2012). The magnitude of global marine species diversity. Curr. Biol..

[2] Mace G.M., Norris K., Fitter A.H. (2012). Biodiversity and ecosystem services: A multilayered relationship. Trends Ecol. Evol..

[3] Barbier E.B. (2017). Marine ecosystem services. Curr. Biol..

[4] Jackson J.B.C., Kirby M.X., Berger W.H., Bjorndal K., Botsford L.W., Bourque B.J., Bradbury R., Cooke R., Erlandson J., Estes J.A. (2001). Historical overfishing and the recent collapse of coastal ecosystems. Science.

[5] Fabricius K.E. (2005). Effects of terrestrial runoff on the ecology of corals and coral reefs: Review and synthesis. Mar. Pollut. Bull..

[6] Burke L., Reytar K., Spalding M., Perry A. (2011). Reefs at Risk Revisted.

[7] Bianchi C.N., Morri C. (2000). Marine biodiversity of the Mediterranean Sea: Situation, problems and prospects for future research. Mar. Pollut. Bull..

[8] Myers N., Mittermeier R.A., Mittermeier C.G., Da Fonseca G.A.B., Kent J. (2000). Biodiversity hotspots for conservation priorities. Nature.

[9] Cuttelod A., García N., Abdul Malak D., Temple H., Katariya V., Vie’ J.-C., Hilton-Taylor C., Stuart S.N. (2008). The Mediterranean: A biodiversity hotspot under threat. The 2008 Review of the IUCN Red List of Threatened Species.

[10] Serena F., Abella A.J., Bargnesi F., Barone M., Colloca F., Ferretti F., Fiorentino F., Jenrette J., Moro S. (2020). Species diversity, taxonomy and distribution of Chondrichthyes in the Mediterranean and Black Sea. Eur. Zool. J..

[11] Stevens J.D., Bonfil R., Dulvy N.K., Walker P.A. (2000). The effects of fishing on sharks, rays, and chimaeras (chondrichthyans), and implication for marine ecosystems. ICES J. Mar. Sci..

[12] Mulas A., Bellodi A., Cannas R., Carbonara P., Cau A., Marongiu M.F., Pesci P., Porcu C., Follesa M.C. (2019). Resource partitioning among sympatric elasmobranchs in the central-western Mediterranean continental shelf. Mar. Biol..

[13] Dulvy N.K., Fowler S.L., Musick J.A., Cavanagh R.D., Kyne P., Harrison L.R., Carlson J.K., Davidson L.N., Fordham S.V., Francis M.P. (2014). Extinction risk and conservation of the world’s sharks and rays. eLife.

[14] Abushaala N.M., Shaibi T., Howaege H.M. (2019). New records of rare species in the Mediterranean Sea (November 2020). Mediterr. Mar. Sci..

[15] Ferretti F., Myers R.A., Serena F., Lotze H.K. (2008). Loss of large predatory sharks from the Mediterranean Sea. Conserv. Biol..

[16] Ferretti F., Osio G.C., Jenkins C.J., Rosenberg A.A., Lotze H.K. (2013). Long-term change in a meso-predator community in response to prolonged and heterogeneous human impact. Sci. Rep..

[17] Follesa M.C., Marongiu M.F., Zupa W., Bellodi A., Cau A., Cannas R., Colloca F., Djurovic M., Isajlovic I., Jadaud A. (2019). Spatial variability of Chondrichthyes in the northern Mediterranean. Sci. Mar..

[18] Walls R.H., Dulvy N.K. (2020). Eliminating the dark matter of data deficiency by predicting the conservation status of Northeast Atlantic and Mediterranean Sea sharks and rays. Biol. Conserv..

[19] Iglésias S.P., Toulhoat L., Sellos D.Y. (2010). Taxonomic confusion and market mislabelling of threatened skates: Important consequences for their conservation status. Aquat. Conserv. Mar. Freshw. Ecosyst..

[20] Iglésias S.P. (2014). Handbook of the Marine Fishes of Europe and Adjacent Waters (A Natural Classification Based on Collection Specimens, with DNA Barcodes and Standardized Photographs)—Chondrichthyans and Cyclostomata.

[21] Ebert D.A., Stehmann M. (2013). Sharks, batoids, and chimaeras of the North Atlantic. FAO Species Catalogue of Fishery Purpose.

[22] Mancusi C., Baino R., Fortuna C., Gil De Sola L., Morey G., Bradai M.N., Kallianotis A., Soldo A., Hemida F., Saad A.A. (2020). MEDLEM database, a data collection on large Elasmobranchs in the Mediterranean and Black seas. Mediterr. Mar. Sci..

[23] Bellodi A., Porcu C., Cau A., Marongiu M.F., Melis R., Mulas A., Pesci P., Follesa M.C., Cannas R. (2018). Investigation on the genus *Squalus* in the Sardinian waters (central-western Mediterranean) with implications on its management. Mediterr. Mar. Sci..

[24] Marongiu M.F., Porcu C., Bellodi A., Cannas R., Cau A., Cuccu D., Mulas A., Follesa M.C. (2017). Temporal dynamics of demersal chondrichthyan species in the central western Mediterranean Sea: The case study in Sardinia Island. Fish Res..

[25] Spedicato M.T., Massutí E., Mérigot B., Tserpes G., Jadaud A., Relini G. (2019). The MEDITS trawl survey specifications in an ecosystem approach to fishery management. Sci. Mar..

[26] Follesa M.C., Carbonara P. (2019). Atlas of the Maturity Stages of Mediterranean Fishery Resources Studies and Reviews.

[27] Follesa M.C., Agus B., Bellodi A., Cannas R., Capezzuto F., Casciaro L., Cau A., Cuccu D., Donnaloia M., Fernandez-Arcaya U. (2019). The MEDITS maturity scales as a useful tool for investigating the reproductive traits of key species in the Mediterranean Sea. Sci. Mar..

[28] Baltosser W.H., Zar J.H. (1996). Biostatistical analysis. Ecology.

[29] Goldman K.J., Musick J.A., Bonfil R. (2005). Age and growth of elasmobranch fishes. Management Techniques for Elasmobranch Fisheries.

[30] Lamarca M.J. (1966). A simple technique for demonstrating calcified annuli in the vertebrae of large elasmobranchs. Copeia.

[31] Stevens J.D. (1975). Vertebral rings as a means of age determination in the blue shark (*Prionace glauca* L.). J. Mar. Biol. Assoc. UK.

[32] Cailliet G.M., Martin L.K., Kusher D., Wolf P., Welden B. (1983). Techniques for enhancing vertebral bands in age estimation of California elasmobranchs. NOAA Tech. Rep..

[33] Bellodi A., Mulas M., Cau A., Sion L., Carbonara P., Follesa M.C. (2019). Chapter 4: Cartilaginous species. Handbook on Fish Age Determination: A Mediterranean Experience. Studies and Reviews.

[34] Porcu C., Bellodi A., Cau A., Cannas R., Marongiu M.F., Mulas A., Follesa M.C. (2020). Uncommon biological patterns of a little known endemic Mediterranean skate, *Raja polystigma* (Risso, 1810). Reg. Stud. Mar. Sci..

[35] Bellodi A., Porcu C., Cannas R., Cau A., Marongiu M.F., Mulas A., Vittori S., Follesa M.C. (2016). Life-history traits of the long-nosed skate *Dipturus oxyrinchus*. J. Fish Biol..

[36] Follesa M.C., Cannas R., Cabiddu S., Cau A., Mulas A., Porcu C., Cau A. (2012). Preliminary observations of the reproductive biology and diet for the Norwegian skate *Dipturus nidarosiensis* (Rajidae) from the central western Mediterranean Sea. Cybium.

[37] Porcu C., Marongiu M.F., Olita A., Bellodi A., Cannas R., Carbonara P., Cau A., Mulas A., Pesci P., Follesa M.C. (2020). The demersal bathyal fish assemblage of the central-western Mediterranean: Depth distribution, sexual maturation and reproduction. Deep. Sea Res. Part I Oceanogr. Res. Pap..

[38] Bellodi A., Mulas A., Carbonara P., Cau A., Cuccu D., Marongiu M.F., Mura V., Pesci P., Zupa W., Porcu C. (2021). New insights into life–history traits of Mediterranean electric rays (*Torpediniformes Torpedinidae*) as a contribution to their conservation. Zoology.

[39] Wallace J.H. (1967). The Batoid Fishes of the East Coast of Southern Africa. Manta, Eagle, Duckbill, Cownose, Butterfly and Sting Rays. Investigational Report.

[40] Last P., White W., de Carvalho M., Séret B., Stehmann M., Naylor G. (2016). Rays of the World.

[41] Serena F., Holtzhausen J., Ebert D.A., Mancusi C. (2015). *Myliobatis aquila*. The IUCN Red List of Threatened Species. https://www.iucnredlist.org/species/161569/124508353.

[42] Rosenberg A., Bigford T., Leathery S., Hill R., Bickers K. (2000). Ecosystem approaches to fishery management through essential fish habitat. Bull. Mar. Sci..

[43] Cau A., Follesa M.C., Moccia D., Bellodi A., Mulas A., Bo M., Canese S., Angiolillo M., Cannas R. (2017). *Leiopathes glaberrima* millennial forest from SW Sardinia as nursery ground for the small spotted catshark *Scyliorhinus canicula*. Aquat. Conserv..

[44] Heupel M., Carlson J.K., Simpfendorfer C.A. (2007). Shark nursery areas: Concepts, definition, characterization and assumptions. Mar. Ecol. Prog. Ser..

[45] Weigmann S. (2016). Annotated checklist of the living sharks, batoids and chimaeras (chondrichthyes) of the world, with a focus on biogeographical diversity. J. Fish Biol..

[46] Ebert D.A., Fowler S., Compagno L. (2013). Sharks of the World.

[47] Kabasakal H. (2006). Distribution and biology of the Bluntnose Sixgill shark, *Hexanchus griseus* (Bonnaterre, 1788) (Chondrichthyes: *Hexanchidae*), from Turkish Waters. Ann. Ser. Hist. Nat..

[48] Sims D.W. (2009). Differences in habitat selection and reproductive strategies of male and female sharks. Sex. Segreg. Vertebr..

[49] Ebert D.A., Compagno L.J., Cowley P. (2006). Reproductive biology of catsharks (Chondrichthyes: *Scyliorhinidae*) off the west coast of southern Africa. ICES J. Mar. Sci..

[50] Porcu C., Marongiu M.F., Follesa M.C., Bellodi A., Mulas A., Pesci P., Cau A. (2014). Reproductive aspects of the velvet belly *Etmopterus spinax* (Chondrichthyes: *Etmopteridae*), from the central western Mediterranean Sea. Notes on gametogenesis and oviducal gland microstructure. Mediterr. Mar. Sci..

[51] Marongiu M.F., Porcu C., Bellodi A., Cannas R., Carbonara P., Cau A., Coluccia E., Moccia D., Mulas A., Pesci P. (2021). Abundance, distribution and reproduction of the data-deficient species (*Squalus blainville*) around Sardinia Island (central western Mediterranean Sea) as a contribution to its conservation. Mar. Freshw. Res..

[52] Sulikowski J.A., Irvine S.B., DeValerio K.C., Carlson J.K. (2007). Age, growth and maturity of the roundel skate, *Raja texana*, from the Gulf of Mexico, USA. Mar. Freshw. Res..

[53] Porcu C., Bellodi A., Cannas R., Marongiu M.F., Mulas A., Follesa M.C. (2014). Life-history traits of a commercial ray, *Raja brachyura* from the central western Mediterranean Sea. Mediterr. Mar. Sci..

[54] Schluessel V., Bennett M.B., Collin S.P. (2010). Diet and reproduction in the white-spotted eagle ray *Aetobatus narinari* from Queensland, Australia and the Penghu Islands, Taiwan. Mar. Freshw. Res..

[55] Skate S. (2021). The IUCN Red List of Threatened Species—Leucoraja circularis.

[56] Du Buit M.H. (1977). Age et croissance de *Raja batis* et de *Raja naevus* en Mer Celtique. ICES J. Mar. Sci..

[57] Carbonara P., Bellodi A., Palmisano M., Mulas A., Porcu C., Zupa W., Donnaloia M., Carlucci R., Sion L., Follesa M.C. (2020). Growth and age validation of the thornback ray (*Raja clavata Linnaeus*, 1758) in the south Adriatic Sea (central Mediterranean). Front. Mar. Sci..

[58] Goldman K.J., Caillet G.M., Andrews A.H., Natanson L.J., Carrier J.C., Musick J.A., Heithaus M.R. (2012). Assessing the age and growth of chondrichthyan fishes. Biology of Sharks and Their Relatives.

[59] Capapé C. (2008). Diet of the angular rough shark *Oxynotus centrina* (Chondrichthyes: *Oxynotidae*) off the Languedocian coast (southern France, north-western Mediterranean). Vie Milieu.

[60] Guallart J., García-Salinas P., Ahuir-Baraja A.E., Guimerans M., Ellis J.R., Roche M. (2015). Angular roughshark *Oxynotus centrina* (Squaliformes: *Oxynotidae*) in captivity feeding exclusively on elasmobranch eggs: An overlooked feeding niche or a matter of individual taste?. J. Fish Biol..

